# Influence of Community and Culture in the Ethical Allocation of Scarce Medical Resources in a Pandemic Situation: Deliberative Democracy Study

**DOI:** 10.2196/18272

**Published:** 2020-03-30

**Authors:** Monica Schoch-Spana, Emily K Brunson, Howard Gwon, Alan Regenberg, Eric S Toner, Elizabeth L Daugherty-Biddison

**Affiliations:** 1 Johns Hopkins Center for Health Security Bloomberg School of Public Health Johns Hopkins University Baltimore, MD United States; 2 Department of Anthropology Texas State University San Marcos, TX United States; 3 Department of Environmental Sciences Bloomberg School of Public Health Johns Hopkins University Baltimore, MD United States; 4 Johns Hopkins Berman Institute of Bioethics Johns Hopkins University Baltimore, MD United States; 5 Division of Pulmonary and Critical Care Medicine School of Medicine Johns Hopkins University Baltimore, MD United States

**Keywords:** pandemic, COVID19, influenza, disaster, preparedness, scarce resources, culture, ethics

## Abstract

**Background:**

Stark gaps exist between projected health needs in a pandemic situation and the current capacity of health care and medical countermeasure systems. Existing pandemic ethics discussions have advocated to engage the public in scarcity dilemmas and attend the local contexts and cultural perspectives that shape responses to a global health threat. This public engagement study thus considers the role of community and culture in the ethical apportionment of scarce health resources, specifically ventilators, during an influenza pandemic. It builds upon a previous exploration of the values and preferences of Maryland residents regarding how a finite supply of mechanical ventilators ought to be allocated during a severe global outbreak of influenza. An important finding of this earlier research was that local history and place within the state engendered different ways of thinking about scarcity.

**Objective:**

Given the intrastate variation in the themes expressed by Maryland participants, the project team sought to examine interstate differences by implementing the same protocol elsewhere to answer the following questions. Does variation in ethical frames of reference exist within different regions of the United States? What practical implications does evidence of sameness and difference possess for pandemic planners and policymakers at local and national levels?

**Methods:**

Research using the same deliberative democracy process from the Maryland study was conducted in Central Texas in March 2018 among 30 diverse participants, half of whom identified as Hispanic or Latino. Deliberative democracy provides a moderated process through which community members can learn facts about a public policy matter from experts and explore their own and others’ views.

**Results:**

Participants proposed that by evenly distributing supplies of ventilators and applying clear eligibility criteria consistently, health authorities could enable fair allocation of scarce lifesaving equipment. The strong identification, attachment, and obligation of persons toward their nuclear and extended families emerged as a distinctive regional and ethnic core value that has practical implications for the substance, administration, and communication of allocation frameworks.

**Conclusions:**

Maryland and Central Texas residents expressed a common, overriding concern about the fairness of allocation decisions. Central Texas deliberants, however, more readily expounded upon family as a central consideration. In Central Texas, family is a principal, culturally inflected lens through which life and death matters are often viewed. Conveners of other pandemic-related public engagement exercises in the United States have advocated the benefits of transparency and inclusivity in developing an ethical allocation framework; this study demonstrates cultural competence as a further advantage.

## Introduction

### Legal and Ethical Preparedness for an Influenza Pandemic

Pandemic readiness and response activities are urgently underway, prompted by certainty about future global influenza outbreaks as well as the human and economic losses suffered during recent epidemics such as with the severe acute respiratory syndrome illness [1-3]. The pandemic of the novel coronavirus that first emerged in late 2019 has also acutely demonstrated the importance of robust preparedness and response systems. Legal and ethical matters have been a principal consideration for pandemic planning, domestically and internationally [4-10]. Of specific concern are public health measures that could adversely affect trade, travel, and economic stability; tip the balance between personal liberty and public wellbeing; or strain people’s sense of justice or fairness [4,5]. Among the legal and ethical issues and dilemmas that the World Health Organization (WHO) first outlined for member states’ consideration when undertaking pandemic preparedness planning are state of emergency declarations, movement restrictions, mass gathering prohibitions, school closures, isolation and quarantine, volunteer licensing and liability, drug and vaccine manufacturer liability, research participant protections, compulsory vaccination, and resource rationing [6].

Allocation of scarce lifesaving resources is as an especially fraught issue. Stark gaps exist between projected human health needs in a pandemic and the current capacity of public health, health care, and medical countermeasure systems. Finite production capacity and delayed vaccine development (ie, 4 months to produce the first doses of a novel pandemic strain vaccine and 6 months to generate a large supply) force the issue of who receives the initial doses [10,11]. What proportions of an antiviral stockpile to commit to treatment and prophylaxis, as well as who to prioritize constitute another dilemma [12]. Likewise, the projected number of US patients requiring an intensive care unit (ICU) bed and mechanical ventilation during a 1918-like pandemic eclipses current capacity by orders of magnitude [13]. In other contexts (eg, African countries with weak economies and endemic malaria and HIV/AIDS), a higher order predicament exists: whether to prioritize a response to a pandemic flu or to hyperendemic diseases [6,14].

### Public Engagement With Pandemic Policies

Common to the current guidance on pandemic ethics—and specifically scarce resource allocation—is the call for public engagement (ie, the broader community’s participation in the policy decisions affecting them) [4-9,15-17]. Pandemic ethicists invoke principles such as transparency, inclusivity, and education and information, citing multiple benefits. Authorities are enjoined to:

1. Open up decisions to scrutiny and articulate clearly the rationale behind specific choices. As a result, the public can be more confident that policies are not capricious, but reasonable, equitable, and in line with community views [4,5,7,9,16,17].
2. Elicit community input early on, especially that of disadvantaged groups. By doing so decision makers can more readily earn public trust, enhance social solidarity, add to a policy’s legitimacy, and discern which approaches are socially acceptable and practically feasible [7-9,15-17].
3. Provide the public with accurate, timely, and understandable information about the pandemic threat, measures for personal protection, and collective actions for readiness and response. This can foster an educated populace—one better able to engage meaningfully with policymakers and play their own part in risk reduction [7,15,16].

Since these injunctions, public engagement in pandemic decision making has evolved beyond mere aspiration; although it has yet to become a widely used practice. Some arguments against it are that democratically elected representatives already represent population preferences, charged issues (eg, scarce resource allocation) could generate public emotion and confusion, and an inability to show how community views (particularly divergent ones) influenced policy could fuel public frustration [18]. Nonetheless, over the last 15 years, members of the public have had some degree of opportunity to inform potentially divisive pandemic policies in the United States [19-40] (see Multimedia Appendix 1 for more details) and elsewhere [41-47]. Often convened by public health agencies, and occasionally by university researchers, private citizens and interested stakeholders have participated in dialogue and deliberation sessions about pandemic dilemmas in which ethical principles (eg, liberty, beneficence, fairness) and technical matters (eg, disease containment, medical treatment) are inextricably bound.

Of the 18 known US pandemic-related public engagement exercises, more than half addressed how to ethically apportion finite lifesaving resources like vaccines, antivirals, intensive care, or personal protective equipment, while about a quarter debated burdensome social distancing measures (Multimedia Appendix 1). Of those discussions focused on scarce resources, nearly half considered prioritized access to intensive care (ie, ICU bed or ventilator). Other countries in which efforts have been made to glean diverse public views on ethically complex decisions during an influenza pandemic include Canada, Australia, New Zealand, and Switzerland [17,41-48]. Most of these studies addressed the allocation of scarce medical resources.

### Cultural Aspects of Pandemic Ethics

An issue bearing upon influenza pandemic ethics in general, and public engagement in particular, has been what regard to give the diverse local contexts and cultural perspectives that shape the experiences of and responses to an otherwise global health threat [14,48]. The WHO ethical guidance for a pandemic highlights the need for international solidarity and shared principles, as well as a recognition that local circumstances and cultural values also influence ethical considerations [16]. In its pandemic ethical guidelines, the Centers for Disease Control and Prevention (CDC) proposes balancing centralized decision making at the federal level and implementation by state and local communities, and it advises consideration toward marginalized communities and groups whose cultural, religious, and other values require sensitivity [9]. Procedural ethics—in particular, bringing affected groups to the table so that their needs and views on a shared health threat are genuinely heard, and they see a policy as having been made fairly even if they may not agree with the final decision—are identified as a key means to respect cultural differences while advancing pandemic preparedness and response [14].

Culture and public engagement have surfaced as relevant issues for pandemic preparedness and planning, in particular among nations that include often marginalized indigenous populations. New Zealand’s ethics guidance asserts that given the disproportionate impact of past pandemics, it is essential to mobilize expertise within Maori communities on how best to address their own situation and needs [17]. Similarly, calls have been made for greater inclusion of Aboriginal and Torres Strait Islander peoples in Australian national, state, and territory pandemic plans so that the heightened risk of these indigenous groups can be mitigated in more culturally appropriate ways [49-51]. Planning experiences with Canada’s First Nations communities and American Indian and Alaska Native communities have also evidenced the value of participatory processes in uncovering the influence of local beliefs, values, and practices upon pandemic health and in strengthening the cultural competence, social acceptance, and practical feasibility of pandemic preparedness and response efforts that affect these groups [52,53].

Alongside in-nation differences, culture is also seen as a force shaping a nation’s or entire region’s pandemic approach. The notion of a “one-size-fits-all” plan for ethically allocating scarce medical resources butts up against divergent sociocultural conditions [14,16]. The higher status accorded to elders within many African societies—including those where the proportion of young children is higher than in other countries—may moderate the importance of the young as a priority group [14]. Among Asian countries where honoring older adults, senior personnel, royalty, and public service staff is a strong norm, and where family ties accord strong obligations, privileged access along social hierarchies and familial lines to nationally stockpiled antivirals is not necessarily seen as unethical [14]. Contrasts made with an American ethos of individualism and wariness toward government include Canada’s communitarian political culture emphasizing peace, order, and good government [54,55]; Australia’s broad embrace of a utilitarian liberal rationale prioritizing others’ safety over individual autonomy [48]; and the United Kingdom’s “Dunkirk spirit” engendering solidarity and public cooperation with state emergency efforts [56]. Noted attitudinal differences between the US and UK populations about pandemic preparedness may also reflect different health care traditions and the greater influence of religious beliefs in the United States [54,56].

### Project Purpose

This US-based study considers the role of community and culture in the ethical apportionment of scarce health resources in a flu pandemic. It builds upon prior work by the authors exploring the values and preferences of Maryland residents regarding how a finite supply of mechanical ventilators ought to be allocated during a severe global outbreak of influenza [37-39]. Our initial aims were to develop, pilot, and apply a deliberative democratic procedure for conducting community forums state-wide that would inform a Maryland framework for the allocation of scarce health care resources in a disaster. An important finding was the need to convene a diverse, regionally varied sample of state residents to capture different ways of thinking about scarcity that local history and place seemed to influence [37,38]. Given the intrastate variation in the themes expressed by Maryland participants, the project team decided to examine interstate differences by implementing the same protocol elsewhere. We wondered if the same core values continue to hold in other areas of the United States, whether variation in ethical frames of reference exists at the US regional level, and what practical implications of sameness and difference may follow for pandemic planners and policymakers.

## Methods

### Study Location, Population, and Recruitment

This research took place in Central Texas. Community members were recruited from two areas: the greater San Antonio metropolitan area in Bexar County (comprising urban and semiurban communities with a high percentage of persons of Hispanic origin) and Colorado County (a rural county to the northeast of San Antonio with a largely white and less wealthy population). English and Spanish speakers over the age of 18 years were recruited in both of these areas through a variety of methods including: flyers posted in libraries, coffee shops, grocery stores, and other public locations; newspaper and radio ads; and social media postings. To ensure a diverse sample, members of underrepresented ethnic, racial, and socioeconomic communities were purposefully overrecruited.

Data collection occurred in-person and lasted approximately 7 hours. Data collection sessions were held on a single weekend day (Saturday in Colorado County and Sunday in San Antonio) in March 2018. Sessions were convened in hotel meeting rooms in centralized locations in each area to maximize accessibility. All participants were provided snacks and lunch and compensated US $100. Institutional review board committees at Johns Hopkins University and Texas State University approved the project methodology.

### Deliberative Democracy Methods

Methodologically, this study used a deliberative democracy process that has been used with other potentially divisive policy decisions [57,58] and that the researchers had previously adapted to facilitate community discussions about the ethical allocation of scarce medical resources in a disaster [37,38]. Deliberative democracy is a qualitative method that provides a structured process through which community members can both learn facts about a public policy matter from experts and explore their own and others’ views in a moderated community forum. As described in the literature, this approach provides opportunities for *knowledge exchange* between experts/policy makers and the public, *democratic accountability* through broad community representation in discussions that relates to the common good, and *innovation* when crowdsourcing generates new insights and solutions to existing problems [57,58].

In this project, the deliberative democracy process involved four distinct steps. First, community members were given details about a pandemic flu scenario and how scarce medical resources could potentially be distributed. This information was provided via an extended background document that participants read beforehand and an expert-led presentation at the start of the forum. Second, community members then met in groups of 4-7 where they engaged in a moderated discussion around the question: “What should we do in situations where there are more patients needing ventilators than there are ventilators to use?” During the discussion, participants were prompted to consider six ethical principles for prioritizing ventilator use: those most likely to survive the current illness; those most likely to live longest; those who have lived the fewest life stages; those with value to others in a pandemic; first come, first served; and a lottery. Third, at the discussion’s end, each group developed 1-2 questions that they then asked to a panel of subject matter experts that included clinicians, ethicists, and disaster experts. Fourth, after this Q and A panel, and a break for lunch, moderated group discussions continued, this time focusing on the question: “Should healthcare providers be allowed to take a ventilator away from one patient who needs it to survive and give it to another patient who also needs it to survive?”

### Data Collection and Analysis

Data were collected in two forms. Trained note takers documented each group discussion on laptops using a template developed for the project. These notes were supplemented by audio recordings made of all of the group discussions, which were later listened to by the note takers to expand their notes and check for accuracy.

Qualitative data resulting from the group discussions was later examined in an inductive manner via thematic analysis. After reading and rereading notes from the group discussions to ensure familiarity with the data, author EB developed a code list by examining the data relating to each ethical principle for prioritizing ventilator use (morning session) and participants’ responses to the question “Should healthcare providers be allowed to take a ventilator away from one patient who needs it to survive and give it to another patient who also needs it to survive?” (afternoon session). This process resulted into an initial codebook that was re-evaluated and revised following subsequent iterations of coding, grouping codes into themes and re-evaluating the resulting coding schema. After two iterations of this process a final codebook of all codes and their corresponding themes was constructed. This final codebook was then used to re-evaluate every set of notes a final time. This process provided the opportunity to check that the coding, including the resulting themes, was an accurate representation of the group discussion data.

In addition to the group discussions, pre- and postsurveys were administered to all participants. The surveys collected information on participants’ demographics and perceptions of the allocation of ventilators during a pandemic flu scenario, opinions on expert and community decision making, and opinions on the deliberative democracy process. For this study, the survey data was used to provide summaries of respondents’ data so that direct comparisons could be made to the Maryland sample. These descriptive statistics were obtained through analysis in SPSS.

## Results

### Participant Demographics

A total of 30 community members participated in 6 discussion forums, 1 in Colorado County and 5 in San Antonio. One of the forums in San Antonio was comprised entirely of health professionals including clinicians, emergency preparedness experts, and public health officials. The separation of these individuals into a professional group was done to ensure that participants in other discussion groups were not influenced by perceived “expert” voices, and to collect data on the views and opinions of professionals whose work experiences afforded them additional insights into the topics discussed.

Participant characteristics are presented in Table 1. Participants ranged in age from 19-80 years, with the majority being 51-75 years of age. Men and women were represented in nearly equal numbers. Two-thirds of the participants identified their race as white, and nearly half of the entire sample noted being of Hispanic, Latino, or Spanish descent (a proportion smaller than the 60% of San Antonio residents and larger than the 30% of Colorado County residents who claimed Hispanic or Latino heritage in 2018). About a third of participants noted never having been married, and a similar proportion said that they had children living at home. Independents and Democrats were represented in roughly equal numbers, followed by Republicans. Religious affiliation was nearly entirely Christian or Catholic. Most participants had a college education or higher, and similar numbers earned US $40,000-$100,000 or over US $100,000.

**Table 1:** Characteristics of community forum participants convened in Central Texas, March 10-11, 2018.

Variable		Participants (n=30), n (%)
**Age (years)**		
	≤25	3 (10)
	26-50	9 (30)
	51-75	16 (53)
	≥76	1 (3)
	Undisclosed	1 (3)
**Sex**		
	Female	15 (50)
	Male	14 (47)
	Undisclosed	1 (3)
**Ethnicity **		
	Of Hispanic, Latino, or Spanish descent	14 (47)
	Not of Hispanic, Latino, or Spanish descent	15 (50)
	Undisclosed	1 (3)
**Race**		
	Black/African American	2 (7)
	White	20 (67)
	Asian	4 (13)
	Native Hawaiian or other Pacific Islander	1 (3)
	Other	2 (7)
	Undisclosed	1 (3)
**Marital status**		
	Never married	9 (30)
	Married	16 (53)
	Divorced/widowed	5 (17)
Children living at home		11 (36.6)
**Political affiliation **		
	Independent	9 (30)
	Democrat	10 (33)
	Republican	6 (20)
	None/other	5 (17)
**Religious affiliation **		
	Christian/Catholic	28 (93)
	Hindu	1 (3)
	Other	1 (3)
College graduate or higher		21 (70)
**Household income (US $)**		
	<40,000	5 (17)
	40,000-100,000	12 (40)
	>100,000	12 (40)
	Undisclosed	1 (3)

It is important to note that the sample was small, consisting of only 6 discussion forums comprised of 30 total individuals. Such a sample size would be prohibitive in survey research; however, the sample size is appropriate to qualitative methods like deliberative democracy where findings are not considered to be generalizable in the traditional sense. The sample was also not representative of Central Texas as a whole; there was a bias toward an urban population and an overrepresentation of highly educated people. At the same time, diversity within the discussion groups in regard to age, education, race and ethnicity, and other factors likely mitigated some of these issues, as respondents within discussion groups spoke with and listened to one another. Regardless, it is important to keep in mind that the findings reported below are suggestive, not generalizable, of Central Texans’ views regarding ventilator allocation and reallocation.

### Broad Themes: Family and Fairness

Throughout the forum discussions in both Colorado County and San Antonio, two general themes emerged in relation to how community members felt that scarce medical resources should be allocated. The first was the importance of family. Respondents repeatedly referred to family—their own and others—in relation to decisions about ventilator use during an emergency. In some instances, this took the form of participants stating that they and others would be willing to sacrifice themselves for their family members, as one participant in Colorado County stated:

If you have a family that comes in, you have a mom, dad, and two kids, the parents are 9 times out of 10 going to tell you to go to the kids first. They’re gonna say, ‘We’re all hurt, but go to my kids, then come back to me’.

Participant CO-5

In other instances, participants argued that familial roles should be an important consideration in making allocation decisions, as a respondent in San Antonio suggested:

We also have to look at their family situation. Are they a mother? I think that may play a little part of it.

Participant SA 5-3

More broadly, however, participants, and particularly the expert group in San Antonio, referred to the importance of the family as a key cultural value in central Texas that must be considered in light of any policy decisions. As one member of this group explained:

This [referring to the six ethical principles] is a medical model but people will be thinking in a very familial way. The medical standpoint is clear, but it will be implemented into a community who are extremely family oriented. They don’t see themselves as ‘one’ they see themselves as a whole family.

Participant SA 5-6

The sentiment was echoed by another member of this group who stated:

In San Antonio, it’s not what the patient wants it’s what the family has to say. They make decisions together.

Participant SA 5-3

Without a familial focus, including involving families extensively in the decision-making processes, these experts, along with members of other forums in San Antonio and Colorado County, suggested that trust between clinicians and the broader community would be lost.

The decision-maker is bigger than the individual. Providers need to make a plan that will make everyone agree on what needs to be done. You need the cooperation of everyone by negotiating instead of telling the family by force because they are going to rebel.

Participant SA 5-3

As a solution, forum participants suggested that community discussions and education, well before an emergency happens, would enable families to discuss and accept policies beforehand.

The second broader theme emerging from forum discussions was the importance of fairness in deciding on a policy and implementing it. Participants in all discussion groups talked about this issue at length. Most participants stated that they had trust in clinicians generally; however, they were not all convinced that providers, or others in decision-making positions, would be infallible or completely unbiased, as one participant in San Antonio explained:

If a doctor is affiliated with a company, they will just do what the company tells them to do rather than do what he truly thinks would be appropriate, and that just does not seem fair to me. Unfortunately, many of us laypeople just don’t have the knowledge needed to know how to react to such situations...

Participant SA 4-2

For allocations to be fair, participants argued that clear criteria need to be developed and followed consistently within and between locations.

Overall there is nothing fair about any of this [allocating scarce resources in an emergency]. So, the only way you can kind of say you’re being fair is to be consistent.

Participant SA 2-4

In addition, multiple participants also suggested that how different populations might be placed at a disadvantage by a given criterion should be considered, as a participant from the expert San Antonio group explained:

What she was saying about where the hospitals are and the distributions of ventilators in hospitals... this easily provides disadvantages because of the distributions of hospitals in the cities. We are talking about social determinacy of health. It’s a major disadvantage for greater populations living in the south and north. First come first serve may look good, but because of the disproportionate (distribution) of hospitals within the city I don’t think it would work.

Participant SA 5-7

To facilitate fairness, forum members suggested several mechanisms that could be put in place before an emergency including a universal medical records system (as this would inform decisions based on who would be most likely to survive a current illness and live the longest) and the redistribution of ventilators so there is equal geographic coverage (both within cities and between counties in the state) based on current population sizes.

### Specific Responses to the Six Ethical Principles

The themes of family and fairness were woven throughout the forum discussions; however, respondents also had specific thoughts on the six ethical principles, which, based on participants’ survey responses (captured postevent), varied in terms of acceptable to not acceptable in the order of: survive current illness; live longer; value to others; fewer life stages; first come, first served; and lottery (Table 2).

Reasons given for favoring those most likely to survive the current illness and those who will live longer were similar. Respondents felt that in terms of saving the most lives, these two principles presented the best options.

I think from a numbers perspective this makes a lot of sense. You get the most survivors.

Participant SA 2-3

It was also noted that successfully treating people in a pandemic situation would possibly be a morale booster, as a participant in San Antonio noted:

If more people are recovering, then that could boost population morale and people could not freak out as much. Hearing good news in a tough time may make this a little bit easier for everyone.

Participant SA 2-5

In terms of concerns, respondents noted that it is impossible for clinicians to be certain of who will survive or live the longest, and that in regards to patients’ health histories (a factor respondents felt would be important in determining who is most likely to live the longest) this criteria might cause patients or their family members to lie in order to “cheat the system”.

Prioritizing those who have value to others in the pandemic—including clinicians and vaccine developers—received mixed responses; although this principle was ranked third based on survey data. In favor of this principle, participants noted the importance of health care workers, especially clinicians with specific specialty training and in medically underserved locations. At the same time, several forum members noted that this principle has great potential for bias (ie, health care providers favoring their colleagues) and that this principle has the potential to open the door for prioritizing others based on things like socioeconomic status.

[I]f we say we are prioritizing health care providers or people who could help, I’m sure that someone could make a case for someone who just happens to make more money than everyone else. They may be more financially able to help the recovery... and squeeze into being considered.

participant SA 2-5

In relation to prioritizing those who have lived the fewest life stages, forum participants repeatedly noted that putting children first was an important cultural value and critical because children represent the future, as a Colorado County resident stated:

You just generally want to help the children, or the younger people, so they can be prosperous and survive. It’s harder to see a baby die, or a young kid. You want to help them as best you can versus someone who has already lived and had experience in life.

Participant CO-5

At the same time, respondents also noted that the differentiation of life stages beyond infancy and childhood are less clear, that the principle does not resolve situations in which the patients being considered are the same age, and that this principle is biased against older adults who have experience and knowledge that should be respected and preserved.

The most unpopular principles in the survey and in the discussions were first come, first served and a lottery. Participants generally agreed that both of these approaches could be objectively fair; however, they also noted that they were the least likely to save lives:

It does not save the most number of lives....But when in a critical situation, one might need to make more choices than just picking a number out of a box.

Participant SA 4-5

Additionally, forum members in both Colorado County and San Antonio noted bias in favor of those who live in urban areas close to hospitals (a factor impinging upon first come, first served) and the social unacceptability of a lottery because it is seen as a form of gambling.

On a moral and religious aspect we’d be leaving everything to luck. Like, are you going to leave life to luck? Are we going to play bingo with my life?

Participant SA 1-6

**Table 2:** Proportion of responses for how often each principle should be used in making allocation decisions across all respondents, with Texas/Maryland comparison.

Principle	Never or rarely		Often or always		
	Texas (n=30), n (%)	Maryland (n=310), n (%)		Texas (n=30), n (%)	Maryland (n=310), n (%)
Survive current illness	2 (6.7)	25 (8.1)		26 (86.7)	220 (71.0)
Survive longest	8 (26.7)	50 (16.1)		15 (50.0)	174 (56.1)
Fewest life stages	9 (30.0)	87 (28.1)		10 (33.3)	93 (30.0)
Value to others	6 (20.0)	65 (21.0)		12 (40.0)	149 (48.1)
First come, first served	12 (40.0)	149 (48.1)		8 (26.7)	65 (21.0)
Lottery	24 (80.0)	254 (81.9)		3 (10.0)	12 (3.9)

### Reactions to Withdrawing a Ventilator to Give to Someone Else

Participants were reluctant at first to say if it was ever acceptable to remove a ventilator from someone who needs it to survive and give it to someone else who also needs it to survive. Some wanted to avoid judgment on such a complex issue; others felt unqualified to make the call. As people struggled to come to terms with the scenario, some ideas proved more supportable than others. Saving lives was a commonly expressed objective. Participants generally agreed that it was acceptable to remove someone from a ventilator if the patient’s health was not significantly improving and if the equipment might ultimately preserve another life: “If a person’s...getting worse and worse...and they’re obviously not going to survive, it makes sense to me to remove the ventilator and give it to someone else who could do better” (Participant SA 4-3). Respondents felt that having an established timeframe would limit the arbitrary reallocation of the ventilators; although they expressed divergent views about the proper timeline.

Some individuals, however, objected to the reallocation of ventilators entirely.

Life is important to everyone. If someone is on a ventilator that means they have a need....I don’t see a reason to ever take it away.

Participant SA 5-2

Fairness emerged as one reason for their objections, as in it would be unfair to remove a ventilator from someone who had already met the criteria to receive one in the first place. When comparing allocation and reallocation scenarios, some individuals expressed the feeling that choosing to give one person a ventilator over another person was different from removing a ventilator from a patient when death would certainly follow. For some participants, playing a role in someone’s death was an action set aside for a high power.

It doesn’t sound as natural. I guess ‘cause you’re already hooked up.... Morally I think of God’s plan to how things should work out. It just... it’s different.

Participant CO-2

Doctors frequently were identified as trusted arbiters in the difficult decision to remove a ventilator.

...[T]he doctor has the last word. In a situation in which you have two car crashes, he’s not going to flip a coin to see who is going to get helped. He makes the decision based on his academic background and the values in the community.

Participant SA 4-2

During deliberations, however, a tension often arose between participants wanting to rely on medical professionals and being worried that mistakes would still be made. Giving doctors access to “more data” and “predictive modeling”, in addition to their own expert opinions, was offered as one way to reduce such mistakes. To foster community faith in health professionals’ reallocation decisions, participants proposed greater transparency: advising the public in advance of a crisis what criteria will be used, involving the patient’s family in the decision making, and alerting patients at the outset about the possibility of ventilator removal: “If I know the criteria going in and out, I think that is fair” (Participant SA 2-3).

A majority of participants did agree that there are situations in which health care providers should remove a ventilator from one patient who needs it to survive and give it to another who also needs it to survive. Nonetheless, a significant portion still remained against or ambivalent about this scenario (Table 3).

**Table 3:** Proportion of responses to the question, “Are there situations in which health-care providers should remove a ventilator from one patient who needs it to survive and give it to another who also needs it to survive?” with Texas/Maryland comparison.

Responses	Texas (n=29), n (%)	Maryland (n=310), n (%)
Yes	18 (62.1)	195 (62.9)
No	4 (13.8)	68 (21.9)
Unsure	7 (24.1)	47 (15.2)

## Discussion

### Principal Findings

In anticipation of extreme health emergencies like a pandemic influenza or COVID-19, authorities at all levels have been developing ethically informed frameworks for the allocation of scarce medical resources. This study’s purpose was to investigate whether core values concerning scare resource allocation exist in different regions of the United States, while considering the practical implications of sameness and difference for emergency preparedness and response policies.

### Comparison of US Public Engagement Findings

Despite distinct geographies and histories, Maryland and Central Texas residents expressed a common, overriding concern about the fairness of allocation decisions and their implementation: What if someone tries to “game the system” (eg, give false medical histories to circumvent a survivability assessment)? What if a clinician makes an error in judgment about who really needs a ventilator? What if a first come, first serve scenario means that people who live in “hospital-poor” city neighborhoods and rural towns miss out on lifesaving equipment? [37]. The two groups similarly advised on how to build up public faith that the burdens of disease and benefits of treatment are equitably distributed: conduct public education in advance, make the criteria for allocation decisions transparent, and coordinate facilities across the state so that the allocation criteria are consistently applied [37].

Uniting on another point, the two states’ participants identified “survive current illness” and “live longer” as the most acceptable ethical principles that should be used “often” or “always” in making allocation decisions (Table 2), with even more Texans embracing survivability. Both groups also expressed a similar degree of moral ambivalence toward the act of withdrawing a ventilator from someone who needs it to give to another person who also needs it (Table 3). Actively revoking life-sustaining support from a dependent patient, even if to help someone more likely to benefit, was, for many Marylanders and Texans, not morally equivalent to withholding the resource in the first place—whether grounded in spiritual matters (eg, only a higher power has the right to determine a person’s time) or a sense of fair play (eg, taking a ventilator away to be allotted to someone was unjustly changing the rules of the game).

The Central Texas findings also resonated with sensibilities expressed in public engagement exercises convened elsewhere on the allocation of scarce medical resources in a pandemic. Withdrawing lifesaving care similarly evoked concern in Seattle and King County, WA [30,31]. Fairness as a rationing principle also prompted the most discussion in Minnesota-held forums [24,26]. Harris County, TX residents equally embraced having an advance allocation plan that could inform doctors’ allocation decisions during a crisis [34]. Along with Central Texas residents, communities from the Gulf Coast, the mid-Atlantic, the Northwest, and the Midwest converged upon the idea of first helping those most likely to survive, thus saving the most lives [28-31,34,37-39]. That certain foundational principles cut across the country’s distinct regions augurs well, in that a set of core values could potentially sustain a productive national conversation and potential policy framework on an ethically complex matter.

### Family-Centered Approaches to an Allocation Framework

The Maryland and Central Texas forums evidenced common views and values, but dissimilarities also emerged. Participants from both states spoke to a willingness to make sacrifices for their kin, namely, a parent’s or grandparent’s desire to give their allotted ventilator to children in their family. Nonetheless, Central Texas deliberants more readily expounded upon *family* as central to the question of how best to allocate scarce medical resources. Survivability was an important clinical consideration, but so too was the social consideration of a patient’s familial role (eg, are they a mother). Moreover, San Antonio participants, particularly the expert group, singled out family as a key local cultural value. Authorities, they argued, who want to connect effectively with local residents and gain their trust in regard to allocation decisions, should approach families, not individuals, as the partner to engage in understanding prioritization rationale, especially in the case of a withdrawal scenario.

The emergence of family during the Central Texas deliberations as a principal lens through which to view life and death matters has a strong regional cultural basis. Social and behavioral researchers have theorized *familismo*, family-centeredness, as a core cultural ideal among Mexican-origin peoples in particular and, to an extent, Hispanics more generally [59]. Features of the *familismo* value system include obligations among family members (nuclear and extended) to provide economic and emotional support, perceptions of kin as a reliable source of help, family as a core aspect of self-identity, and consultation and conformity with family regarding personal decisions and actions [60]. Mexican Americans tend to view life-threatening illness as a problem for the entire family and health care decisions as a collective matter, in contrast to the privileging of autonomy in Anglo-American medical ethics [61]. These sentiments were particularly present among the groups convened in San Antonio, a city in which a majority of residents claim Mexican heritage; however, family was also a recurrent theme among the study’s predominantly white participants from rural Colorado County. This is likely indicative of a cultural bias within the region where the family is a central institution [62,63].

### Producing and Practicing a Culturally Competent Allocation Policy

Ethicists working on pandemic influenza preparedness have outlined the benefits that public engagement principles of transparency, inclusivity, and education and information afford. When authorities subject their decisions and rationale to public scrutiny, communities can see that policy choices are neither arbitrary nor dismissive of local sensibilities [4,5,7,9,16,17]. Seeking the public’s counsel—including that of marginalized, underrepresented groups—on potential policy directions can foster greater trust in authorities, strengthen legitimacy of decisions, and enable successful implementation [7-9,15-17]. Informing the public in advance about pandemic risk as well as individual and collective ways to manage it can generate a populace who is better equipped to exercise responsibility for community well-being [7,15,16].

A majority of public engagement exercises convened in the United States around pandemic influenza, including those held in Maryland and Texas by the study team, have worked to embody the principles of transparency and inclusivity. They have done so primarily by involving participants who represent the jurisdiction’s larger, heterogeneous population (Multimedia Appendix 1). This sampling approach lends greater credibility to claims of a democratic process, that is, genuinely including the people’s voice in policy discussions. Moreover, most pandemic-related public engagement initiatives have endeavored to discern prevailing community values for policy makers to consider. From Maryland to Texas, areas of agreement abound both on an allocation framework’s substance (eg, “survive current illness” and “live longer” as the most acceptable principles) and its implementation (eg, keep planning transparent, apply framework consistently) [37-39].

At the same time, public deliberation forums have also revealed instances of divergent thinking; although this has been a lesser analytic focus for most other conveners (Multimedia Appendix 1). Holding 15 public engagement forums across Maryland, the study team heard residents speak about fairness in concrete, local, place-based terms, not in the abstract [37,38]. Residents of historically underserved Baltimore city neighborhoods worried about being passed over again; citizens of outlying, rural districts feared that city dwellers would get a disproportionate share of ventilators, just like state revenues. Cognizant of these concerns, state and local health authorities can communicate before, during, and after an emergency about ventilator allocation with greater empathy and in terms that are salient for specific communities.

Contrasting the Maryland and Central Texas forums demonstrates another kind of difference: US regional and ethnic cultures that can affect the development, implementation, and communication of an ethical allocation framework. Foreseeing such an issue, the CDC’s pandemic ethical guidelines advise striking the right balance between centralized decision making at the federal level and implementation at state and community levels where local situations and sensitivities can be better assessed and addressed [9]. *Familismo* has greater relevance in the southwestern United States than in northern, midwestern states where few Hispanics reside. In a state with many Mexican heritage residents and a wider regional and cultural emphasis on family, Texas health authorities may find themselves having to weigh family as a prioritization principle more heavily than peers in other US regions. Another public engagement exercise that oversampled for Spanish speakers found, for instance, that Hispanics prioritized children and pregnant women at much higher rates than non-Hispanics [31]. In addition, the varying religious compositions of US regions may shape the ethical principles that matter most to local communities in relation to allocation matters. Christianity, for instance, represents 59% of the religious composition of adults in New York City, in contrast to 77% in Texas [64].

*Familismo* also has implications for framework implementation and communication. If a state like Texas develops a framework to standardize allocation decisions, then community-level health authorities and clinicians could localize how it is procedurally administered and conveyed to the wider community. This could help create a more culturally competent approach to scarce resource allocation. In respecting *familismo*, for instance, health facilities could adopt communication strategies that strengthen cross-cultural competency among critical care staff [65]. Moreover, health facilities could establish a process that gives family members a chance to confer among themselves when the patient no longer benefits from mechanical ventilation and withdrawal is called for. The emphasis within the *familismo* value system upon the collective and duty to the whole also provides a salient moral frame through which local authorities could communicate effectively about the need for developing and applying an allocation framework, and the importance of taking proactive measures that would help delay the implementation of an allocation framework in the first place, such as strengthening medical surge capacity.

### Limitations

Claims regarding Central Texas regional culture are limited by a small sample size and a bias toward an urban population with a high percentage of persons of Mexican origin. In this segment of Texas, cultural geographers recognize a convergence of four distinct cultural identities: the Anglos of Southern tradition, the Catholics of strong European heritage, the Hispanos, and the African Americans [62]. Due to financial constraints we were not able to employ random sampling. That small groups of participants may not capture broader community interests and views is a frequent critique of deliberative methods [66]. The sample may not represent the region’s four cultural streams, but intragroup diversity (Table 1) and the Maryland contrast nonetheless facilitated a limited investigation of sameness and difference. The time required to attend the deliberative sessions (7 hours) may also have introduced nonparticipation bias.

### Conclusions

Ethicists working on pandemic influenza have proposed that procedural ethics—namely, bringing together diverse communities to weigh in on a policy decision that may affect them so that authorities fairly consider their views and needs—are an important means to respect cultural differences while advancing the common good of stronger preparedness and response systems [14]. Conveners of pandemic-related public engagement exercises in the United States have similarly advocated the benefits of transparency and inclusivity in the development of an ethical framework to guide the allocation of finite medical resources such as mechanical ventilators during a public health catastrophe (Multimedia Appendix 1). The Maryland and Central Texas comparison that reveals *familismo* as a distinctive regional and ethnic core value, moreover, demonstrates that public engagement efforts can ultimately enhance the cultural competence of an ethical allocation framework’s development, implementation, and communication [52,53].

## Appendix

Multimedia Appendix 1 Public Engagement Initiatives for Influenza Pandemic Preparedness, 2005-2017.

## Abbreviations

CDC: Centers for Disease Control and Prevention
ICU: intensive care unit
WHO: World Health Organization.
