# Supplementary material for: Influence of Community and Culture in the Ethical Allocation of Scarce Medical Resources in a Pandemic Situation: Deliberative Democracy Study
Source: J Particip Med. 2020 Mar 30;12(1):e18272. doi: 10.2196/18272 (PMC7141421; doi:10.2196/18272)
Supplement: Multimedia Appendix 1 [file jopm_v12i1e18272_app1.docx]

Multimedia Appendix. Public Engagement Initiatives for Influenza Pandemic Preparedness, 2005-2017.

| **Refs** | **Date/Locale(s)** | **Convening Bodies** | **Issue(s) Presented** | **Engagement Mode/(N)** | **Finding Highlights** |
| --- | --- | --- | --- | --- | --- |
| 1-2 | 2005  Georgia Massachusetts Nebraska  Oregon  Washington, DC | CDC in collaboration with 13 other groups; facilitated by The Keystone Center | Allocation of scarce medical resources – federal priorities (vaccines) | Deliberation and voting on pandemic vaccination goals at national stakeholder meeting (N = 50) and community consultation/ feedback sessions (N = 266) | Top priority immunization goals were assuring society’s functioning and reducing individual deaths and hospitalizations – unaligned with prior expert views |
| 3 | 2006  Massachusetts | Massachusetts Department of Health and Harvard School of Public Health Center for Public Health Preparedness | Allocation of scarce medical resources (antivirals);  Prioritization of critical care;  Government seizure of private assets;  Provider duty to care | 2 stakeholder deliberation sessions including state residents and providers (N = 30) that informed multidisciplinary working group discussions | Consumers and providers agreed prioritization to occur at state level, with aggressive communication to providers and public; both groups opposed maximizing life-years saved by providing key critical care *only* to patients with expected survival > 6 months |
| 4 | 2006-2008  Nassau County, NY  Las Cruces, NM  Milwaukee ,WI  Hendersonville, NC  Washington, DC | CDC and DHS in collaboration with 15 other groups; facilitated by The Keystone Center | Allocation of scarce medical resources – federal priorities (vaccines) | Dialogue/deliberation sessions (N= 500) and stakeholder meetings (N = 80) to elicit ideas on 10 goals (pre-guidance) and then to suggest revisions to guidance | Priority vaccination recipients were persons essential to pandemic response, essential to community functioning, at-risk due to jobs, and children. |
| 5 | 2006  Seattle, WA  Syracuse, NY  Lincoln, NE  Atlanta, GA  Washington, DC | CDC, ASTHO, and 13 other groups; facilitate by The Keystone Center | Imposition of potentially disruptive social distancing measures | Dialogue/deliberation sessions and stakeholder meeting (N = 259) | Support was expressed for measures to keep sick persons at home, change work patterns and schedules, and cancel large gathering, accompanied by ideas on how to mitigate negative social effects |
| 6-8 | 2007-2009  Minnesota | Minnesota Pandemic Ethics Project – led by Minnesota Center for Health Care Ethics and University of Minnesota Center for Bioethics, for the Minnesota Department of Health (MDOH) | Allocation of scarce medical resources (antivirals, vaccines, N95/surgical masks, and ventilators) | Panel-generated, resource-specific frameworks vetted via written comments (N = 116 respondents) and deliberated during 2 community forums (N = 200) and 9 small groups (N = 125) | Prompting the most discussion were 2 issues: the role of fairness as a rationing criterion and how to consider age as a non-clinical rationing criterion |
| 9 | 2008  Boston, MA  Kansas City, KS | ASTHO, CDC, and 11 other partners | Clarification of definition for “at-risk populations”; Identification of unmet needs | Dialogue/deliberation sessions with at-risk individuals (N = 123) and representatives of organizations serving them (N = 21) | Planners were advised to reach at-risk individuals not connected to community networks and to understand systemic barriers at-risk people face |
| 10-11 | 2008  Southeast Michigan | University of Michigan Schools of Public Health and Medicine | Imposition of potentially disruptive social distancing measures;  Allocation of scarce medical resources (antivirals, vaccines, ventilators) | Focus groups with educational session and facilitated discussion among local residents (N = 37) | School/business/religious closings were seen to create economic burdens, deprive people of solace, and have differential impacts  Utilitarian arguments (do what’s best for society, help those most likely to survive) colored allocation recommendations |

| **Refs** | **Date/Locale(s)** | **Convening Bodies** | **Issue(s) Presented** | **Engagement Mode/(N)** | **Finding Highlights** |
| --- | --- | --- | --- | --- | --- |
| 12-13 | 2009  Seattle and King County, WA | Public Health – Seattle and King County aided by advisory committee | Allocation of scarce medical resources (ICU beds, ventilators) and alterations in standards of care | 3 dialogue/deliberation sessions among diverse citizens (N = 123) and 1 with stakeholders (N = 30) | Overall support for importance of survivability as treatment prioritization criterion, with goal to treat as many people as possible even if standards of care lowered; equity came to eclipse societal role as priority allocation principle |
| 9,14 | 2009  Franklin County,  Cuyahoga County, OH | Ohio Department of Health, Ohio State University Center for Public Health Practice, Susan Podziba & Associates, and 5 other groups | Imposition of potentially disruptive social distancing measures | 2 community dialogue/ deliberation sessions (N = 116) and 2 stakeholder meetings (N = 49) | Communities selected issues of primary concern – Franklin: closures of schools, childcare, and malls/movie theaters; Cuyahoga: impacts of social distancing on religious practices |
| 2,9 | 2009  Waianae Coast,  Honolulu,  Hawaii | Hawaii Department of Health with 4 groups assisting including Hawaii Research Center for Futures Studies | Allocation of scarce medical resources (vaccines) | Local TV programs and online, interactive computer game based on pandemic influenza; 2 community forums (N = 33+) and infrastructure stakeholder meeting (N = 19) | At community forums, most highly rated values were “Ensure that public health and safety are priorities in the event of a flu pandemic” and “Result in the greatest good for the greatest number of people” |
| 2,9 | 2009  Cambridge,  Springfield,  Massachusetts | Massachusetts Department of Health and steering committee involving federal, state and local stakeholder; facilitated by contractor | Communication before/during pandemic, including about altered standards of care | 3 community meetings employing deliberation format (N = 135+) | Most important social values, pre- and post-meeting, were “ensure that everyone has the best chance of survival after getting the flu” and “ensure the public health and safety are priorities in the event of a flu” |
| 2,9 | 2009-2010  Winnebago Tribe,  Ponca Tribe,  Lincoln Indian Center,  Chadron Native American Center,  Nebraska | Nebraska Department of Health in conjunction with tribal entities and local health departments | Imposition of potentially disruptive social distancing measures – i.e., modification of tribal/cultural gatherings | 5 community meetings with information and discussion sections (N = 92+) | Most highly rated value pre-meeting was “support everyone getting the same treatment;” most highly rated post-meeting was “ensure that public health and safety are priorities in the event of a flu pandemic;” citizens generally thought burials, wakes, and memorials should not be cancelled |
| 15 | 2009  10 HHS Regions | CDC in collaboration with The Keystone Center, the University of Nebraska Public Policy Center, and WestEd | Implementation of a mass vaccination program during 2009 H1N1 pandemic | 10 face-to-face public meetings with discussion and electronic polling (go-easy, be moderate, go-full throttle), 2 web dialogue sessions, and 1 stakeholder meeting (total N = 1,095) | Majority of meeting participants favored moderate level of preparedness for a mass vaccination program, assuming impacts comparable to seasonable influenza |
| 16 | 2011  Harris County, Texas | Harris County Public Health and Environmental Services, with assistance from The Keystone Center and Epidemiology Monitor | Allocation of scarce medical resources (antivirals, vaccines, ventilators) | 8 dialogue/deliberation sessions among general public (N = 606) and 1 dialogue/ deliberation session among stakeholders (N = 30) | Top vaccine priorities were health care workers (HCWs) and persons are high risk due to medical condition; top antiviral priorities were HCWs and persons at high risk due to age group; advance ventilator allocation plan seen as highly important and likelihood of recovery as most important criterion |

| **Refs** | **Date/Locale(s)** | **Convening Bodies** | **Issue(s) Presented** | **Engagement Mode/(N)** | **Finding Highlights** |
| --- | --- | --- | --- | --- | --- |
| 17 | 2011  Albany,  Long Island,  Syracuse, Westchester, Buffalo,  New York City,  New York | New York State Task Force on Life and the Law and New York State Department of Health | Allocation of scarce medical resources (ventilators) | 9 focus groups | Detailed findings are unknown |
| 18 | 2012  Ft. Benton, MO  Chattanooga, TN  Los Angeles, CA | Institute of Medicine on behalf of the CDC, in partnership with local public health and community organizations | Acceptability of alternative strategies for distribution and dispensing of antivirals | Small group dialogue/deliberation sessions (N = 232) | Few regional differences emerged; broad support noted for public health authorities to develop alternative strategies to help people gain antiviral access in a pandemic and for a “layered” approach using multiple strategies |
| 19-21 | 2012-2014  Maryland | XXX | Allocation of scarce medical resources (ventilators) | Community forums using deliberative democracy methods (N = 324) | Saving the most lives and saving the most life-years were more preferred allocation principles; participants’ values sometimes diverged from expert guidance; themes diverged over state regions |
| 22 | 2016-2017  Illinois | Chicago and Illinois Departments of Public Health; Chicago Healthcare System Coalition for Preparedness and Response | Allocation of scarce medical resources | Community engagement sessions (9 in 2016, 20 in 2017) | Detailed findings are unknown |

**References**

1. The Keystone Center. Citizen voices on pandemic flu choices: A report of the public engagement pilot project on pandemic influenza. 2005. http://ncdd.org/rc/wp-content/uploads/PEPPPI_FINALREPORT_DEC_2005.pdf. Accessed October 31, 2019.
2. Bernier RH, Wills-Toker C. Case abstracts of a multi-year, multi-project public engagement initiative to better inform governmental health policy decisions. J Particip Med. May 22, 2014. https://participatorymedicine.org/journal/evidence/case-studies/2014/05/22/case-abstracts-of-a-multi-year-multi-project-public-engagement-initiative-to-better-inform-governmental-public-health-policy-decisions/.
3. Levin D, Cadigan RO, Biddinger PD, Condon S, Koh HK. Altered standards of care during an influenza pandemic: identifying ethical, legal, and practical principles to guide decision making. Disaster Med Public Health Prep. 2009 Dec;3 Suppl 2:S132-40. doi: 10.1097/DMP.0b013e3181ac3dd2.
4. University of Nebraska Public Policy Center. Evaluation of Public Engagement Project on Pandemic Influenza Vaccine Prioritization - Phase I: Evaluation of Public and Stakeholder Input. Lincoln, NE; University of Nebraska Public Policy Center; February 2008. http://ppc.unl.edu/wp-content/uploads/2008/02/Pandemic-Infuenza-Evalaution-Report.pdf. Accessed October 31, 2019.
5. The Keystone Center. The Public Engagement Project on Community Control Measures for Pandemic Influenza Findings and Recommendations from Citizen and Stakeholder Deliberation Days. Keystone, CO: The Keystone Center; May 2007. http://ppc.unl.edu/wp-content/uploads/2007/05/FinalReportMay2007.pdf. Accessed October 31, 2019.
6. Garrett JE, Vawter DE, Prehn AW, DeBruin DA, Gervais KG. Ethical considerations in pandemic influenza planning. Minn Med. 2008 Apr;91(4):37-9.
7. Garrett JE, Vawter DE, Gervais KG, Prehn AW, DeBruin DA, Livingston F, Morley AM, Liaschenko J, Lynfield R. The Minnesota Pandemic Ethics Project: sequenced, robust public engagement processes. J Participat Med. 2011 Jan 19; 3:e6. https://participatorymedicine.org/journal/evidence/research/2011/01/19/the-minnesota-pandemic-ethics-project-sequenced-robust-public-engagement-processes/.
8. Vawter DE, Garrett JE, Gervais KG, Prehn AW, DeBruin DA, Tauer CA et al. For the Good of Us All: Ethically Rationing Health Resources in Minnesota in a Severe Influenza Pandemic. St. Paul, MN: Minnesota Center for Health Care Ethics and University of Minnesota Center for Bioethics; 2010. http://www.health.state.mn.us/divs/idepc/ethics/ethics.pdf. Accessed October 31, 2019.
9. University of Nebraska Public Policy Center. Evaluation of Public Engagement Demonstration Projects for Pandemic Influenza. Lincoln, NE; University of Nebraska Public Policy Center; May 2010. http://ppc.unl.edu/wp-content/uploads/2010/05/P5-Report-FINAL.pdf. Accessed October 31, 2019.
10. Baum NM. Resource Allocation in Public Health Practice [dissertation]. Ann Arbor, MI: University of Michigan; 2010.
11. Baum NM, Jacobson PD, Goold SD. "Listen to the people": public deliberation about social distancing measures in a pandemic. Am J Bioeth. 2009 Nov;9(11):4-14. https://doi.org/10.1080/15265160903197531.
12. Li-Vollmer M. Health care decisions in disasters: engaging the public on medical service prioritization during a severe influenza pandemic. J Participat Med. 2010 Dec 14; 2:e17. https://participatorymedicine.org/journal/evidence/case-studies/2010/12/14/health-care-decisions-in-disasters-engaging-the-public-on-medical-service-prioritization-during-a-severe-influenza-pandemic/.
13. Public Health: Seattle & King County. Public Engagement Project on Medical Service Prioritization During an Influenza Pandemic [Internet]. Public Health: Seattle & King County; September 29, 2009. www.kingcounty.gov/healthservices/health/preparedness/~/media/health/publichealth/documents/pandemicflu/MedicalServicePrioritization.ashx. Accessed October 31, 2019.
14. Podziba S, Sachs A, Pearsol J. Planning for a Pandemic: Findings and Recommendations from Ohio Residents and Stakeholders - The Ohio Pandemic Influenza Public Engagement Demonstration Project. Susan Podziba & Associates and the Center for Public Health Practice at The Ohio State University's college of Public Health; October 2009. http://www.cidrap.umn.edu/sites/default/files/public/php/452/452_socialdistancing_report.pdf. Accessed October 31, 2019.
15. Keystone Center. The Public Engagement Project on the H1N1 Pandemic Influenza Vaccination Program - Final Report. Keystone, CO: Keystone Center; September 2009. http://ppc.unl.edu/wp-content/uploads/2009/09/Final-H1N1-Report-Sept-30-2009-Keystone.pdf. Accessed October 31, 2019.
16. Harris County Public Health and Environmental Services. The Harris County Public Engagement Project on Pandemic Influenza [Internet]. Harris County Public Health and Environmental Services. https://www.keystone.org/wp-content/uploads/2015/08/072911-Harris-County-TX-Pandemic-Influence-Engagement-Project-Report.pdf. Accessed October 31, 2019.
17. New York State Task Force on Life and the Law, New York State Department of Health. Ventilator Allocation Guidelines. November 2015. https://www.health.ny.gov/regulations/task_force/reports_publications/docs/ventilator_guidelines.pdf. Accessed October 31, 2019.
18. IOM (Institute of Medicine). Public Engagement on Facilitating Access to Antiviral Medications and Information in an Influenza Pandemic: Workshop Series Summary. Washington, DC: National Academies Press; 2012.
19. Daugherty Biddison EL, Gwon HS, Schoch-Spana M, Regenberg AC, Juliano C, Faden RR, Toner ES. Scarce Resource Allocation During Disasters: A Mixed-Method Community Engagement Study. Chest. 2018 Jan;153(1):187-195. https://doi.org/10.1016/j.chest.2017.08.001
20. Daugherty Biddison EL, Gwon H, Schoch-Spana M, Cavalier R, White DB, Dawson T, Terry PB, London AJ, Regenberg A, Faden R, Toner ES. The community speaks: understanding ethical values in allocation of scarce lifesaving resources during disasters. Ann Am Thorac Soc. 2014 Jun;11(5):777-83. https://doi.org/10.1513/AnnalsATS.201310-379OC
21. Daugherty Biddison EL, Faden R, Gwon HS, Mareiniss DP, Regenberg AC, Schoch-Spana M, Schwartz J, Toner ES. Too many patients…A framework to guide statewide allocation of scarce mechanical ventilation during disasters. Chest. 2018 Oct 11. pii: S0012-3692(18)32565-0. doi: 10.1016/j.chest.2018.09.025. [Epub ahead of print]
22. The Chicago Department of Public Health (CDPH) and the Illinois Department of Public Health (IDPH) are cosponsoring a series of community engagement meetings around the state entitled "Healthcare in Disaster Situations: Your Voice Matters." (meetings in 2016-2017). http://www.dph.illinois.gov/news/healthcare-disaster-situations-your-voice-matters https://chscpr.org/crisis-standards-of-care-planning/
